# Daily Monitoring of Emotional Responses to the Coronavirus Pandemic in Serbia: A Citizen Science Approach

**DOI:** 10.3389/fpsyg.2020.02133

**Published:** 2020-08-19

**Authors:** Selka Sadiković, Bojan Branovački, Milan Oljača, Dušanka Mitrović, Dejan Pajić, Snežana Smederevac

**Affiliations:** Department of Psychology, Faculty of Philosophy, University of Novi Sad, Novi Sad, Serbia

**Keywords:** COVID-19, emotional reactions, RRST, the state of emergency, citizen science

## Abstract

The COVID-19 pandemic, a health emergency with international consequences, has brought serious impact on all aspects of society and affects not only health and economy, but psychological functioning and mental health as well. This research was conducted in order to examine and further our understanding of emotional reactions to the ongoing pandemic. Change in emotional reactions during the pandemic and relations with specific pandemic related behaviors and personality traits from the revised Reinforcement sensitivity theory were explored. The research was conducted in Serbia for 35 days while the country was in a state of emergency, as a citizen science project. Out of the 1526 participants that joined the study, 444 (67% female) had measures for all five weeks. Longitudinal changes in four emotional states during the pandemic were examined: worry, fear, boredom, and anger/annoyance. Results indicate a decrease in all four emotional states over time. The biggest decrease was present in case of worry, followed by fear and boredom. Regression analysis showed that personality dimensions, as well as behavioral responses in this situation significantly predicted emotional reactions. Findings revealed the Behavioral activation system was significantly related to worry, fear and boredom, Fight with boredom and anger, and the Behavioral inhibition system with anger. Adherence to protection measures, as well as increased exposure to the media, had significant positive relations with worry and fear. These results indicate that both stable characteristics and specific pandemic-related behaviors are significantly related to emotional response during the pandemic.

## Introduction

The outbreak of Coronavirus disease 19 (COVID-19) has led to a global health crisis that has hit the population of many countries. As, at the time of writing, this crisis was still ongoing and involves many unknowns, the magnitude of its consequences has been difficult to predict. Nevertheless, it is evident that it has affected many aspects of life – health, economic, but also mental health and psychosocial functioning. The sources of altered psychosocial functioning in such a situation are, on the one hand, linked to the very presence of the threat of infection and its potentially dangerous outcomes, and on the other, to the various measures taken in most countries to prevent the spread of the infection.

During a health crisis caused by a pandemic of a viral disease, the potential and invisible threat may enhance anxiety-related responses, such as worry. Uncertainty and perceived lack of control in such circumstances, resulting from the nature of the threat, increases anxiety (e.g., Taha et al., 2014). Moreover, when the infection is caused by a novel virus, people tend to rate the threat as greater than in cases of known infections (Hong and Collins, 2006). In addition, repeated exposure to infection-related information, whether coming from the media or social networks, can lead to heightened stress responses (Rubin et al., 2010; Garfin et al., 2020), but also to a certain level of confusion due to ambiguity of information regarding risk-assessment and precautionary measures.

After the COVID-19 reached pandemic proportions, measures have been taken in order to control it. Apart from recommendations regarding protective behaviors (e.g., keeping distance, hand hygiene, avoiding touching faces, wearing gloves and masks), these measures in most countries involve varying forms of physical distancing from other people, reducing contacts, and consequently changing habits and usual behaviors. Persons who potentially have come into contact with the infection are asked to stay in isolation at their homes or quarantine facilities in order to reduce the risk of infecting other people. The others are usually advised to avoid leaving their homes if not necessary, and in some countries quarantine is introduced as a global measure, regardless of possible previous exposure to the coronavirus. Studies on the effects of quarantine during epidemics suggested that people tend to experience increased frustration and boredom during isolation, which, together with distress due to risk perception, inadequate supplies and financial loss, may lead to confusion, anger and post-traumatic stress symptoms (Hawryluck et al., 2004; Johal, 2009; Brooks et al., 2020). Also, compliance with the measure is lower if the rationale for it is not understood (Reynolds et al., 2008).

The first recorded case of COVID-19 in Serbia was on March 6, 2020. Ten days later, a state of emergency was declared in the country. Universities, schools and kindergartens have stopped working. Classes for younger children have been organized through special TV stations, and university classes through various distance learning online platforms such as Moodle, Zoom, etc. Many people have been working from their homes, most stores and facilities have been closed. Persons over 65 have been banned from leaving their homes, except on weekends from 4 to 7 a.m. for the purchase of basic groceries. From 5 p.m. until 5 a.m. the whole population was forbidden to leave homes. Starting March 29, people were not allowed to leave their homes during weekends, from Friday afternoon till Monday morning. The slight loosening of measures has begun at the end of April.

**FIGURE 1:**
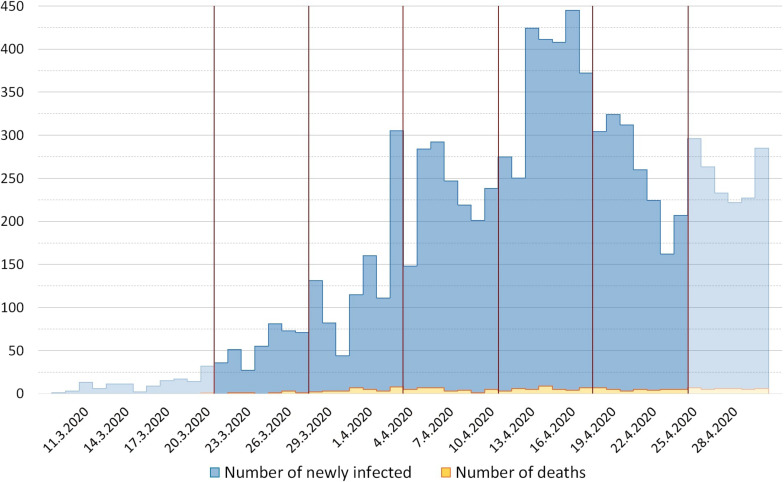
Daily numbers of newly infected persons and deaths due to COVID-19 in Serbia.

Figure 1 provides information on the daily numbers of infected persons in Serbia. Vertical lines indicate 5 weeks covered by the survey presented in this paper.

So far the results of research on the psychological impacts of the COVID-19 epidemic in China have indicated some factors that contribute to the levels of distress during this health crisis. The results of the study which included participants from the general population in China have suggested that the adherence to the precautionary measures and accurate knowledge about COVID-19 were associated with lower levels of stress, anxiety and depression (Wang et al., 2020). Other findings indicated that stressors including worries about economic influence and delays in academic activities, as well as effects on daily life and the lack of social support, predicted higher levels of anxiety in college students in China; living in urban areas, living with parents and stable family income were shown to be protective factors against anxiety, whereas gender was not linked to the level of anxiety (Cao et al., 2020). Results of a nationwide study of psychological distress in China revealed that participants between 18 and 30, and those older than 60, females, higher-educated, and residents of the region of China that was most affected by corona infection, reported higher levels of distress, and findings also indicated a decrease in distress levels over time (Qiu et al., 2020). Prevalence of post-traumatic stress symptoms in Hubei province of China was 7%, with women reporting higher symptoms regarding re-experiencing, negative alterations in cognition and mood, and hyper-arousal (Liu et al., 2020).

The individual responses to health crises can also stem from some stable personality characteristics since they influence the way one perceives a situation and reacts to it. Previous studies suggested that personality traits predict differences in behavioral and affective response to epidemic. For instance, the study conducted during H1N1 epidemic in Turkey indicated that recommended protective behaviors, but also avoidance behavior, were linked to higher Impulsive sensation seeking (Gaygisiz et al., 2012), and Xie et al. (2011) found that the level of anxiety during Severe Acute Respiratory Syndrome (SARS) epidemic in China was linked to pessimism and Mazza et al. (2020) found out that female gender and negative affect are associated with higher levels of anxiety, stress and depression during COVID19 emergency in Italy. However, the results regarding the role of personality in epidemic-related behaviors and reactions seem to have been pretty scarce so far. Although a body of literature on psychological responses to pandemic is based on a five-factor model (e.g., Carvalho et al., 2020; Kroencke et al., 2020; Zettler et al., 2020), there are growing findings that the revised reinforcement sensitivity theory (rRST; Gray and McNaughton, 2000) may provide a relevant framework for studying behavior during epidemics. The rRST emphases the emotional and motivational tendencies that drive attention to environmental signals, and manifest in the human behavior and cognition (e.g., Corr and Krupić, 2017), which may be especially important for understanding responses to health crisis situations (e.g., Bacon and Corr, 2020) and processing health messages (e.g., Shen and Dillard, 2007). The rRST emphasizes the impact of neurophysiological factors on individual differences in behavioral patterns in reaction on (dis)incentives of various kinds (Gray and McNaughton, 2000; Corr and McNaughton, 2012) and proposes three emotional-motivational systems responsible for approach or avoidance behavior in situations that contain signals of reward and punishment/threat (Gray and McNaughton, 2000): the Behavioral activation system (BAS) responsible for reactions to all appetitive stimuli; the Behavioral inhibition system (BIS) defined as the basis for the processing of conflicting stimuli; and the Fight/Flight/Freeze system (FFFS) comprises defensive reactions to all aversive stimuli. Moreover, the BIS is related to the emotion of anxiety and is more focused on anticipated/potential, not immediate, threats. In contrast, the FFFS is related to the emotion of fear, which is triggered by actual threat, and can result in confrontation (defensive aggression), attempt to escape, or cessation of reactions with the aim to evade danger (e.g., Gray and McNaughton, 2000). Fear controls flight, freezing, and defensive fighting behaviors.

Gray and McNaughton (2000) argued that two broad clusters in defensive behavior as a reaction on danger/threat represent the action of two brain systems, one controlling anxiety and the other fear, and it is possible to distinguish between those mechanisms throughout defensive direction: ones particularly prone to fear tend to avoid threat, whereas those who tend to orientate toward threat should be particularly prone to anxiety (e.g., Perkins and Corr, 2006). Humans typically selected fight responses in scenarios describing clear threats, but risk assessment in the case of ambiguous threats. Low fight predicts the tendency to orient away from threats, especially for women, since men scoring high on fight may be prone to a confrontational style of reaction to threatening situations (MacLaren et al., 2010). Since the coronavirus implies an invisible threat, the distinction between anxiety and fear postulated by the rRST makes this model useful in the context of examining emotional responses to the current pandemic. Moreover, recent rRST based research of the perspective on concerns and intention to self-isolate during coronavirus pandemic in the United Kingdom, has shown that both BAS and FFFS personality traits being involved in concerns about coronavirus (Bacon and Corr, 2020). Also, research has shown that negative emotions in response to the current pandemic predict adaptive public health-compliant behavior change, such as hand washing or social distancing (Harper et al., 2020). In other words, functional fear can be a protective factor in coping with danger.

Previous research has shown that the general parameters of monitoring many natural and social phenomena can be accurately obtained in citizen science projects (Haag, 2005; Newman et al., 2010). The principles of citizen science created by the European Citizen Science Association (2016) indicate the need to involve members of the general public in scientific endeavors that contribute to new knowledge or understanding important phenomena. Citizens can, if they wish, participate in several stages of the scientific process, such as the development of a research question, the creation of research methods, the collection and analysis of data, and the dissemination of results. Adequate motivation of citizens is an integral part of the success of citizen science projects, since the small number of participants or the dropout of participants during the project can lead to its termination or failure. Participation in citizen science projects can be based on different levels of engagement (Haklay, 2013), from the extreme, in which scientists and volunteers actively participate in all stages of the project, to the level in which citizens only participate in data collection. Peoples who contribute to different stages of a project, from problem definition to data collection and analysis of results, usually participate because of a strong interest in the project topic rather than specific profits. In this research, citizen scientists were invited to participate in different stages of research, as it is a globally important phenomenon that has influenced the need for all citizens to provide different types of contributions. In this, the first citizen science psychological research in Serbia, citizens gave suggestions for some of the questions, collected data and disseminated the results on social networks. Apart from the students who participated in the research for course credit and promoted it by motivating their relatives, friends, and colleagues to participate, members of the various NGOs have also significantly contributed to the promotion of the research and offered some useful suggestions on the improvement of research methodology. Additionally, members of the research team made several media appearances in order to present preliminary findings and further promote the research. One of the most valuable contributions in recruiting new participants was from the Center for the Promotion of Science of the Serbian Ministry of Education, Science, and Technological Development.

In this study, we examined factors contributing to the emotional responses to the threat of coronavirus infection and isolation due to a pandemic. Based on previous research (Xie et al., 2011; Gaygisiz et al., 2012; Bacon and Corr, 2020; Cao et al., 2020; Carvalho et al., 2020; Harper et al., 2020; Mazza et al., 2020; Qiu et al., 2020; Wang et al., 2020), it is assumed that emotional responses to a pandemic may be related to different factors, both basic dispositions and behaviors specific for the state of emergency. We expect BIS, Flight and Freeze to be associated with anxiety and fear related responses, and BAS and Fight primarily with reactions to isolation. We were also interested in whether emotional states were associated with behaviors specific for the state of emergency, since emotional states can trigger certain behaviors, but behaviors can also induce certain emotional states. While personality traits can be viewed as predictors of emotional responses in various situations, the relationship between behaviors and emotional states in this study was viewed solely from the point of view of a potential correlation, which does not imply an assumption about the direction of the influence. We assumed that behaviors such as following the pandemic-related news in the media and adherence to the recommended precautionary measures are relevant to all emotional reactions, while active work from home, organizing daily routines and engaging in hobbies are relevant to reactions to isolation.

## Materials and Methods

### Sample

During the 5 weeks, a total of 18,478 participant responses were collected from, with a mean average of 527.94 responses per day. The whole sample was comprised of 1,526 participants from Serbia. There were 889 participants in the first week of the research, 885 active participants during the second week, 698 during the third week, 639 during the fourth week and 595 during the fifth week. In total 444 participants provided measures for all 5 time points. The examination was anonymous and no personal information that could identify participants was collected. More information about the sample is given in Supplementary Tables (Supplementary Appendix A). The Ethics Committee of the Department of Psychology at the University of Novi Sad approved the study and the certificate can be found at the following link: http://psihologija.ff.uns.ac.rs/etika/?odobreno=202003171031_OCx7.

### Procedure and Citizen Science

A custom web application was developed for participants to join the study. For each participant, random code was generated which they used to access different surveys and questionnaires. The code was a 13–17-character long string containing randomly ordered letters and digits. The web application was optimized to save anonymized personalized code for each participant using cookies in order to minimize the possibility of error by participants. In the case of the participants recruited by the students, only the principal investigator had information about the passwords that students have assigned to their participants. The anonymity of participants was protected and it allowed students to receive adequate curriculum points. All questionnaires were administered using the Google Forms platform. Data from March 21 up to April 24 were used in the presented research. Four types of forms were administered during the research. The first battery of questionnaires was administered once participants joined the study. After providing informed consent, participants provided various sociodemographic information and responded to several questionnaires including the RSQ. Daily surveys (second form) were administered from Monday to Saturday each week. The third form was a weekly survey administered every Sunday and the fourth type was a monthly survey administered on March 31.

Citizen scientists actively participated in all phases of the research. For example, questions related to substance abuse during a pandemic were suggested by citizen scientists. They actively worked to promote the research, engage the respondents and motivate them to complete the questionnaires on a daily basis. The results of the survey were regularly available on the research website, social networks and media, and citizen scientists contributed to their dissemination. The list of citizen scientists and institutions that supported the research is in the acknowledgment.

### Measures

#### Personality Traits

The Reinforcement Sensitivity Questionnaire (RSQ; Smederevac et al., 2014) is a 29-item questionnaire comprising of five scales that correspond to five systems of rRST (Gray and McNaughton, 2000) and contains 29 items: BIS - Anxiety (7 items, α = 0.77), BAS - Impulsivity (6 items, α = 0.720), Fight (Aggression), Flight (Avoidance) and Freeze (Panic) system (with 5 items each, α = 0.776, α = 0.586, and α = 0.771, respectively). Items are presented on a 4-point Likert scale (from 1 = completely disagree to 4 = completely agree).

#### Responses to Coronavirus and Isolation

These surveys, administered daily, weekly or monthly, assessed how participants were handling the COVID-19 pandemic and the state of emergency in Serbia through assessment of their affective and behavioral responses to the situation. Questions for assessing emotional response (administered daily) in this research are: “Are you occupied with thoughts of the coronavirus today?”, “How afraid are you that you will be infected with the coronavirus today?”, “How bored were you today?”, and “To what extent are you angry, annoyed or aggressive today?”. These questions represented the levels of worry, fear, boredom and anger/annoyance of the participants and were measured using a 5-point Likert scale.

Questions for assessing behaviors specific for the state of emergency were: “I wear protective masks and gloves, to avoid close contact with people in order to protect myself and others,” measured using 5-point Likert scale, “I regularly follow the news about coronavirus on TV, online or through other media,” “I have organized my daily routine,” “I devote time to activities I usually like (reading, listening to music, watching movies, knitting, exercise…)” and “I actively study/work from home” measured using a 3-point scale (Yes, No, and It is not relevant for me). Questions about protective measures were administered on a monthly basis, while other questions concerning behaviors were measured on a weekly basis. These questions assessed the level of structure and organization of participants’ lives.

### Data Analysis

All analyses were performed in SPSS 21 statistical software (Ibm Corp, 2012). In order to compare how worry, fear, boredom and anger/annoyance levels of the participants changed through time repeated measures ANOVA (RM ANOVA) were used. In total 4 RM ANOVA analyses were run, one for each variable (worry, fear, boredom and anger/annoyance items). Data from Responses to coronavirus and the isolation concerning worry, fear, boredom and anger were averaged to 5 measures. Since the first day of the survey was Saturday measures were split weekly from Saturday to Friday. First period (T1) was from March 21 to 27, second period (T2) was from March 28 to April 3, third period (T3) was from April 4 to 10, fourth (T4) period was from April 11 to 17 and the last period (T5) was from April 18 up to 24. Bonferroni corrected *post hoc* tests were used in order to compare differences between individual measurements.

Hierarchical multiple regression was applied in order to examine how specific behaviors and personality traits are related to emotional reactions to pandemic. In total four regression models were run. Measures of worry, fear, boredom and anger/annoyance, averaged from 5 measures previously described, were used as criterion variables. Predictors in the first step of analysis were rRST personality traits: BIS, BAS, Fight, Flight and Freeze. Predictors in the second step of the analyses were behaviors specific for the state of emergency – protection (measured on March 31), media, daily routine, hobby and study/work from home (measured on April 12). For predictors measured on a 3-point scale, “It is not relevant for me” response was removed and predictors were used in binary format (yes, no). All effect sizes were interpreted according to Cohen’s (1988). The supplementary data and data instructions for this article are publically available online at OSF platform: https://osf.io/vejn9/.

## Results

### Descriptive Statistics

Basic descriptive statistics parameters, for all measurement points and all predictors and criterions variables, are shown in Supplementary Appendix B. In general, there were significant gender differences on worry, fear and anger/annoyance but there were no differences on boredom measures. As time passed it seems that gender differences became minimal as there were no gender differences at all in the last week (T5). On all measures with significant gender difference female participants had higher scores compared to male participants, which indicates that women tended to experience negative emotions during the pandemic more intensely compared to men. Age was significantly correlated with worry, fear and boredom in all time points, but it was only weakly negatively correlated to anger in T1, T4, and T5. Older participants had a tendency to worry more than younger participants and were more fearful. On the other hand, age was negatively correlated to boredom. All statistically significant correlations between emotional states and age were significant at *p* < 0.01, except between age and Anger on T4 which was significant at *p* < 0.05. Correlation between RSQ dimensions were all statistically significant at *p* < 0.01. The highest and positive correlation was between BIS and Freeze, while the lowest and negative correlation was between Fight and BIS. Correlations between behaviors related to pandemic were in most cases low in the intensity and were not statistically significant. The relationships between emotional responses were low to medium intensity, positive and statistically significant in all cases. Correlations between mentioned measures are shown in Supplementary Appendix D. Reliability analysis (Supplementary Appendix D), suggested that reliability was in the range from good to excellent, for all used measures.

### Repeated Measures ANOVA

**FIGURE 2:**
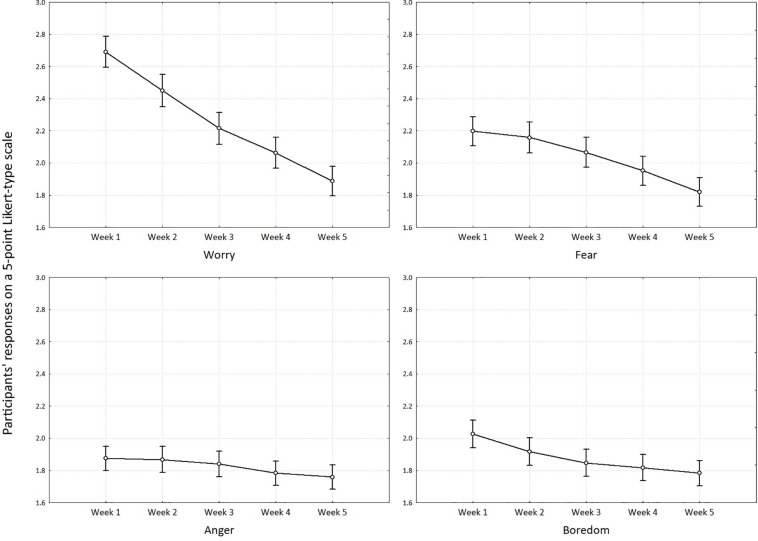
Least squares means for worry, fear, anger and boredom at five time points.

Results of RM ANOVA indicated that change over time was significant for each measure: worry [*F*(1772) = 199.92, *p* < 0.001, *i*^2^_*p*_ = 0.311], fear [*F*(1768) = 60.51, *p* < 0.001, *i*^2^_*p*_ = 0.120], boredom [*F*(1772) = 18.49, *p* < 0.001, *i*^2^_*p*_ = 0.040], and anger [F(1772) = 4.54, *p* < 0.01, *i*^2^_*p*_ = 0.010]. In line with Cohen’s (1988), effect size for worry and fear is large, for boredom was medium, while for anger is small. Results are shown in Figure 2.

Bonferroni *post hoc* tests are shown in Supplementary Appendix C. Worry was consistently higher in earlier measures in comparison to the later ones. The constellation of results is nearly identical for fear, with an exception for T1 and T2 where no significant differences were found. There were no statistical differences between T2 and T3, T3 and T4, T3 and T5 and T4 and T5 for boredom, while all other pairs of comparisons were significantly different. This indicates that even though boredom slowly declined through time more than a week was needed in order for decline to be statistically significant. For anger, there were only two statistically differences between T1 and T5 and T2 and T5.

### Hierarchical Multiple Regression

The results of the set of hierarchical multiple regression analyses are presented in Table 1. VIF parameters, which ranged from 1.07 to 1.94, indicated that multicollinearity was not present between predictor variables. Results suggested that personality traits and behaviors specific for the state of emergency could explain a significant percentage of the emotional reactions, from 7.8% for anger up to 12.2% for worry. For the worry, fear and boredom step 2, which includes personality traits and behaviors, was significantly better than step 1 which includes only personality traits. Only for the anger, step 2 was not significantly better in contrast to step 1. Worry and Fear were significantly and positively related to adherence to protective measures and exposure to news about COVID-19 and negatively related to BAS. Boredom was significantly and positively related to BAS, Fight, and Freeze and negatively related to daily routine and protection. Anger was positively and significantly related to BIS and Fight.

**TABLE 1:** Hierarchical multiple regression analysis for dimensions of the behaviors during isolation and RSQ used to predict emotional response at the beginning of the pandemic (*N* = 456).

**Variable**	**Worry**				**Fear**				**Boredom**				**Anger**			
	**β**	**95% CI**		***p* level**	**β**	**95% CI**		***p* level**	**β**	**95% CI**		***p* level**	**β**	**95% CI**		***p* level**
		**Lower**	**Upper**			**Lower**	**Upper**			**Lower**	**Upper**			**Lower**	**Upper**	
Step 1																
BIS	0.102	–0.004	0.208	0.059	0.098	–0.015	0.211	0.089	0.088	–0.001	0.054	0.089	0.130	0.044	0.216	0.003
BAS	–0.105	–0.189	–0.022	0.013	–0.136	–0.225	–0.048	0.003	0.089	0.019	0.013	0.003	0.000	–0.068	0.068	0.997
Fight	0.084	0.004	0.164	0.040	0.062	–0.023	0.147	0.155	0.081	0.013	0.019	0.155	0.143	0.077	0.208	0.000
Flight	0.082	–0.007	0.170	0.072	0.090	–0.004	0.185	0.062	0.029	–0.046	0.443	0.062	0.004	–0.068	0.077	0.906
Freeze	–0.052	–0.155	0.051	0.320	–0.063	–0.172	0.047	0.261	0.085	–0.001	0.053	0.261	0.069	–0.015	0.152	0.105
	*R* = 0.220**				*R* = 0.226**				*R* = 0.248**				*R* = 0.297**			
	Adjusted *R*^2^ = 0.038**				Adjusted *R*^2^ = 0.041**				Adjusted *R*^2^ = 0.051**				Adjusted *R*^2^ = 0.078**			
	*R*^2^ change = 0.038**				*R*^2^ change = 0.041**				*R*^2^ change = 0.051**				*R*^2^ change = 0.078**			
Step 2																
BIS	0.082	–0.021	0.185	0.117	0.081	–0.029	0.191	0.150	0.077	–0.010	0.165	0.084	0.125	0.038	0.212	0.005
BAS	–0.091	–0.173	–0.010	0.028	–0.123	–0.210	–0.036	0.006	0.091	0.021	0.160	0.011	0.000	–0.069	0.068	0.991
Fight	0.068	–0.009	0.146	0.084	0.047	–0.036	0.130	0.267	0.082	0.015	0.148	0.016	0.143	0.078	0.209	0.000
Flight	0.062	–0.025	0.148	0.160	0.071	–0.022	0.163	0.134	0.031	–0.042	0.105	0.403	0.002	–0.071	0.075	0.951
Freeze	–0.043	–0.143	0.056	0.393	–0.056	–0.163	0.051	0.306	0.091	0.006	0.176	0.037	0.071	–0.013	0.155	0.098
Protection	0.133	0.056	0.209	0.001	0.135	0.052	0.217	0.001	–0.074	–0.140	–0.009	0.026	–0.030	–0.095	0.035	0.363
Media	0.203	0.118	0.289	0.000	0.194	0.102	0.286	0.000	0.023	–0.051	0.096	0.545	0.029	–0.043	0.102	0.431
Daily routine	–0.015	–0.104	0.074	0.745	0.001	–0.094	0.096	0.985	–0.123	–0.199	–0.047	0.002	–0.063	–0.138	0.012	0.099
Hobby	–0.024	–0.137	0.089	0.680	–0.040	–0.161	0.082	0.521	0.057	–0.040	0.154	0.249	0.012	–0.083	0.108	0.800
Study/work from home	–0.042	–0.123	0.039	0.306	–0.025	–0.111	0.062	0.576	–0.050	–0.120	0.019	0.154	0.007	–0.061	0.076	0.833
	*R* = 0.349**				*R* = 0.337**				*R* = 0.322**				*R* = 0.310**			
	Adjusted *R*^2^ = 0.102**				Adjusted *R*^2^ = 0.093**				Adjusted *R*^2^ = 0.084**				Adjusted *R*^2^ = 0.078**			
	*R*^2^ change = 0.084**				*R*^2^ change = 0.052**				*R*^2^ change = 0.033**				*R*^2^ change = 0.000			

*p < 0.05; **p < 0.01.

## Discussion

In this study, we examined emotional responses to the potential threat of coronavirus infection and isolation during 5 weeks of the pandemic. Since most people in this situation have changed specific behaviors and habits, the second objective was to examine the contribution of basic personality traits and new habits, developed during the pandemic, to emotional reactions. The rRST, as a theory that integrates the characteristics of a situation and biological mechanisms of response to a situation, represented an appropriate theoretical framework. This is a citizen science study, in which participants evaluated the pandemic coping strategy on a daily basis.

The most important result of this study is that worry and fear of possible coronavirus infection gradually decreased over 5 weeks. Worry usually arises in a potentially dangerous situation, such as the ubiquitous threat of corona infection. A special feature of this situation is “invisible” danger with no clear indication that the threat has been avoided. At the onset of the pandemic, worry was more pronounced, while a gradual decrease indicated less uncertainty and a stronger feeling of control of everyday life. Higher worry is significantly associated with low impulsivity as well as with regularly focusing attention on the media and information about the prevalence of coronavirus and adhering the preventive measures, such as wearing gloves and social distancing (Table 1). The results are in line with previous findings suggesting that repeated exposure to information related to crisis is connected with a higher level of distress (Rubin et al., 2010; Garfin et al., 2020), but not consistent with the results which suggested that the adherence to precautionary measures is associated with lower levels of distress (Wang et al., 2020).

Similar results were obtained regarding fear of infection over time. The finding that fear is generally less pronounced than worry is in accordance with previous research, showing that anxiety and fear have a different physiological basis and occur in different situations (Gray and McNaughton, 2000). While anxiety occurs as a reaction to potential danger, fear is a reaction to a real danger. Fear decreased after 5 weeks of a pandemic and it is important to emphasize that the predictors of fear and worry are identical. Fear is also associated with low impulsivity and behaviors such as exposure to the media and adhering the preventive measures.

The finding that higher-BAS individuals are less afraid and less worried is not entirely in line with the findings of Bacon and Corr (2020), possibly due to some contextual differences (e.g., data in United Kingdom were collected when no restrictions by government were yet imposed), or to different measures of rRST traits used in the two studies. This is an important result, indicating that the pandemic has provoked a variety of reactions. Expectations that high BIS people will be more worried or afraid during the pandemic have not been confirmed. Instead, a significant predictor of Worry and Fear is low BAS, indicating that lower worry and fear turned out to be characteristics of people who are impulsive and more responsive to reward signals. Prediction both of worry and fear through a low BAS can be interpreted as a connection between impulsivity and lack of functional anxiety, which indicates the possibility that BAS regulates complex reactions to sudden situations and unconditional stimuli, for which there are no previously developed patterns of behavior. Namely, previous studies have shown that BIS and FFFS predict anxiety both in the domain of self-assessment (Ignjatović et al., 2013) and in experimental conditions (Ranelović et al., 2018). However, the coronavirus pandemic represents a completely new and unexpected threat, and it is possible that it provoked the activation of a system that regulates reactions to novel situations, such as BAS. Therefore, it is possible that the coronavirus outbreak contributed to the development of functional anxiety, as an adaptive response to a new situation, which has an important role in searching for behavioral patterns that can contribute to facing the threat, while impulsiveness appears as a significant predisposition for risky behaviors. In other words, the approaching and reward-oriented behavior accompanied by the absence of fear or worry in this situation may reflect a tendency toward risky behavior, especially since it is followed by non-compliance with preventative measures.

The finding that women tend to respond with more intense worry and fear than men is in line with most of the previous results (Liu et al., 2020; Qiu et al., 2020) and is probably related to the generally higher intensity of emotional experience in women (Grossman and Wood, 1993). More pronounced worry in the older participants, which was noted in previous research as well (Qiu et al., 2020), could be related to the knowledge of an increased risk of complications from COVID-19 in the elderly.

A major challenge during a pandemic is adhering to preventive measures that require social distancing and isolation. Our results confirm previous findings that boredom and anger are frequent reactions to quarantine (Hawryluck et al., 2004; Johal, 2009; Brooks et al., 2020). The finding that younger subjects exhibit a higher degree of boredom than older ones might reflect a different degree or type of change in daily life due to pandemic in young and older adults. Nevertheless, both boredom and anger gradually decrease, but with a different pattern than fear and anxiety. Namely, the experience of boredom decreased during weeks 1 and 2 of the research and hit a low point that did not go below as there are no significant differences for week 3 onward (Supplementary Appendix B). The relatively low initial experience of boredom indicates the possibility that the pandemic has provoked the engagement of psychological and behavioral resources to adopt new habits, related to changed everyday life circumstances. Further decline in already low boredom is likely due to the adoption of new strategies for structuring time. Namely, boredom is associated with BAS, Fight, Freeze, lack of protection and reduced usual commitments and activities (Table 1). This is a very interesting result, since it matches the personality traits that contribute to boredom. Namely, apart from BAS, which is usually associated with a tendency to sensations seeking and risky behaviors (e.g., Chase et al., 2017), Fight contributes as well, which can be manifested through a tendency to reject rules. In other words, boredom can represent a type of aggressive resistance to a situation with strict rules and prohibitions. Freeze refers to cognitive blockage due to impending threat (Smederevac et al., 2014). In this context, it is possible that Freeze may contribute to the occupation of cognitive resources by negative emotions, which affects the lack of both initiative and active structuring of time; lack of organized daily routine is the most important predictor of boredom. This result indicates the importance of daily routine for mental health. Structuring time through daily routine can enhance the experience of purpose, self-efficiency and provide cognitive and emotional gratification. Therefore, this result is crucial to understanding coping with isolation, pointing to a strategy that can be controlled and that can enhance emotional responses.

Anger is an emotional reaction that has shown the greatest stability over the 5 weeks of the study, since there were significant differences in the degree of its expression over time only for the first and second week in contrast to last, fifth week (Supplementary Appendix B). Anger was the least pronounced of all emotions and may reflect the general distribution of individual differences in aggression or the Fight system, which represents the tendency to display aggressive behavior as a response to threat (Gray and McNaughton, 2000). This result is significant, since anger is the only emotional reaction associated merely with stable personality traits. Namely, anger is associated with higher levels of Fight and BIS, without the contribution of specific, pandemic related behaviors (Table 1). Obviously, an increase in tension can contribute to aggressive reactions. Both coronavirus threat and isolation contribute to increasing tension, to which otherwise aggressive individuals respond with more frequent or intense anger, which may also be a reaction to helplessness due to a lack of control over a dangerous situation.

The results of this study indicate that worry and fear have an important role in coping with dangerous situations, such as coronavirus pandemic, since they mobilize resources for facing threat. While the situation is unfamiliar, people are finding new patterns of behavior, which causes tension and uncertainty as they are unsure of the success of the new strategies. Over time, the experience of controlling the situation increases and the tension decreases. This is an adaptation strategy, indicating a tendency of people to modify behaviors in accordance with negative circumstances. After 5 weeks, the coronavirus pandemic was no longer a new situation, people slowly adjusted, less worried and afraid. This result is consistent with previous findings that functional fear and negative emotions in response to the current pandemic predict adaptive public health-compliant behavior change (Harper et al., 2020).

Another important implication of these results is that personality traits significantly shape emotional responses during isolation. Although the pandemic has important specificities, it should not be overlooked that people’s reactions reflect their stable, previously learned patterns and strategies. Also, media exposure and lack of daily routine are the basic prerequisites for negative emotional reactions during isolation. Specifically, people who have structure of the daily routine engagement experience less negative emotions, such as boredom. This finding has important implications for treatment design and mental health prevention during a pandemic.

Finally, these results have important theoretical implications for further empirical support for the rRST. It is possible that the role of BAS in responding to unconditional stimuli has previously been underestimated. Despite our expectation that BIS and Flight will shape emotional reactions to the coronavirus pandemic, they have not shown relevant contributions. It is possible that the threat caused by the corona virus was universal, provoking worry and fear among all citizens, which contributed to the reduction of individual differences on BIS and Flight. In other words, perhaps all people were mostly worried, not just those who were otherwise prone to such reactions. Differently, the activity of the BAS is probably provoked by the suddenness of the situation, Fight is provoked by the limitations of preventive measures, while Freeze’s activity is a consequence of preoccupation with negative emotions, which blocked resources for more constructive behavior during self-isolation. Future research should focus on testing the hypothesis that BAS regulates the response to a sudden threat, in the direction of examining its role in the lack of functional worry.

These results should be treated with caution, as certain limitations may affect their generalization. First, participation in this study was voluntary and there is a possibility that our sample meets the WEIRD (Western, Educated, Industrialized, Rich, and Democratic) sample criteria (Henrich et al., 2010). Therefore, we cannot be sure if these participants represent the whole population, since they represent, at best, the features of volunteers. Although this limitation could be applied to virtually all psychological studies, especially during a pandemic when only online contacts are allowed, it is important to keep in mind that it could affect the structure of the sample and generalization of results.

In addition, not all subjects began participation on the same day, since they enrolled during the fifth week of the study. Therefore, the drop out of the sample is large, since we included only respondents who participated in the first week in this study to meet the criteria for repeated measurements. This limitation did not affect the findings of this study. Namely, participants that had measurement on only one time point and those that had all measurements were compared and there were no systematic differences (Supplementary Appendix C). Moreover, due to the correlational design of the study, definite conclusions about the nature of some relationships, particularly those between emotional states and specific behaviors, could not be drawn. It might be that people with certain dispositions are more likely to both engage in specific activities and to experience certain emotions, but it is also possible that some behaviors tend to induce, or to further increase, emotional responses to a situation such as pandemic.

Despite the limitations, these results have an important implication, since they support the previous findings reporting boredom and frustration during isolation (Brooks et al., 2020); gender differences in baseline levels of negative emotions due to quarantine measures (Liu et al., 2020); increased anxiety and worry in the first stages of virus epidemic (Taha et al., 2014); but also a decrease in measured distress levels over time (Qiu et al., 2020).

Finally, a significant merit of this study was participation of citizen scientists, who gave contributions to psychological science and, through participation in research, actively structured their time, which is one of the most important protective factors in coping with crisis situations.

## Data Availability Statement

The datasets presented in this study can be found in online repositories. This data can be found here: https://osf.io/vejn9/.

## Ethics Statement

The studies involving human participants were reviewed and approved by Faculty of Philosophy, University of Novi Sad, Serbia. The ethics committee waived the requirement of written informed consent for participation.

## Author Contributions

SSm designed the research with inputs from DM, DP, BB, SSa and MO. DM and SSa carried out the literature searches and screening and any discrepancies were discussed with SSm. DP and BB organized online data collection and SSm implemented citizen science project strategy. MO carried out the data extraction and analysis with the inputs from BB. SSm wrote the first draft of the discussion with input from DM, DP, BB, MO, and SSa. All authors contributed to the article and approved the submitted version.

## Conflict of Interest

The authors declare that the research was conducted in the absence of any commercial or financial relationships that could be construed as a potential conflict of interest.
