# Supplementary material for: Daily Monitoring of Emotional Responses to the Coronavirus Pandemic in Serbia: A Citizen Science Approach
Source: Front Psychol. 2020 Aug 19;11:2133. doi: 10.3389/fpsyg.2020.02133 (PMC7466566; doi:10.3389/fpsyg.2020.02133)
Supplement: Supplementary file 1 [file Table_1.pdf]

Supplementary

Appendix A.

Sample characteristics

First part of the sample was collected by second-year students in the Psychology program at the University of Novi Sad, Serbia. Each student had the task to invite 2 to 3 members of the family and 3 friends or relatives to participate in the study. Participation was voluntary, and each participant provided informed consent. Second part of the sample were Serbian citizens who voluntarily joined the survey, following invitation on social networks, media and civil society organizations. There were 519 participants collected by students and 1,007 volunteers from the general population.

Among those that had measurements on all time points 301 (67.8%) were female, and the mean age of the sample was 30.04 (SD = 14.26) years (Figure 2). Among those that actively participated in all five time points there were 164 students (36.9%), 124 (27.9%) participants with high school education, and 132 (29.7%) participants with college education. Around 5% of participants had elementary school education or PhD. 430 participants provided information about their material status. Vast majority of the sample (86.7%) reported their material status as mediocre (185; 43%) or good (188; 43.7%), 8.6% (37) of the sample reported having very good material status while less than 5% reported their material status as bad (19) or very bad (2).

Table A.1. Distribution of participants' age and gender

		Age								
		11-20	21-30	31-40	41-50	51-60	61-70	71-80	81-90	Total
A	Males	65	160	37	35	37	4	1	1	340
	Females	222	506	185	162	90	18	2	1	1186
	Total	287	666	222	97	127	22	3	2	1526
B	Males	39	56	4	18	21	4	0	1	143
	Females	109	103	16	35	30	6	1	1	301

	Total	148	159	20	53	51	10	1	1	444

A – Total sample; B – Sample of participants with responses at all five time points

Table A.2. Distribution of participants' level of education

		Level of education					
		Primary	Secondary	Student	Higher	MA/PhD	Total
A	Males	1	100	90	105	44	340
	Females	4	173	330	476	203	1186
	Total	5	273	420	581	247	1526
B	Males	1	56	43	38	5	143
	Females	3	68	121	94	15	301
	Total	4	124	164	132	20	444

A – Total sample; B – Sample of participants with responses at all five time points

Table A.3. Distribution of participants' material status on the scale from 1 (very bad) to 5 (very good)

		Material status						
		1	2	3	4	5	X	Total
A	Males	1	9	75	55	12	188	340
	Females	2	13	129	150	28	864	1186
	Total	3	22	204	205	40	1052	1526
B	Males	1	7	70	52	11	2	143
	Females	1	12	115	136	26	11	301
	Total	4	124	164	132	20		444

A – Total sample; B – Sample of participants with responses at all five time points; X – No response

Table A.4. Distribution of the size of participants' living place

		Size of participants' living place			
		Small	Medium	Large	Total
A	Males	41	58	241	340
	Females	135	180	871	1186

	Total	176	238	1112	1526
B	Males	17	32	94	143
	Females	59	67	175	301
	Total	76	99	269	444

A – Total sample; B – Sample of participants with responses at all five time points

Appendix B.

Descriptive statistics for used variables

	M	SD	SK	Ku	R	Gender[a]		Age[b]
						Male	Female	
T1 worry	2.77	1.08	0.34	-0.64	.929	2.58	2.84**	.188**
T1 fear	2.21	1.00	0.42	-0.68	.938	2.04	2.27**	.169**
T1 boredom	2.14	1.06	0.78	-0.11	.855	2.08	2.15	-.306**
T1 anger	1.97	0.96	1.01	0.45	.789	1.77	2.04**	-.104**
T2 worry	2.61	1.13	1.22	0.93	.760	2.38	2.68**	.201**
T2 fear	2.18	1.03	0.73	-0.04	.897	2.05	2.22*	.148**
T2 boredom	1.98	1.04	0.67	-0.25	.898	1.94	1.99	-.244**
T2 anger	2.00	1.02	0.91	0.28	.903	1.84	2.05*	-.001
T3 worry	2.24	1.06	1.07	0.65	.954	2.04	2.31**	.227**
T3 fear	2.05	1.00	1.21	1.01	.951	1.91	2.11*	.199**
T3 boredom	1.85	0.92	0.80	0.11	.893	1.79	1.87	-.281**
T3 anger	1.90	0.90	0.91	0.16	.832	1.77	1.95*	-.072
T4 worry	2.05	1.02	0.97	0.19	.956	1.90	2.11*	.204**
T4 fear	1.95	0.97	1.12	0.81	.958	1.79	2.01*	.174**
T4 boredom	1.81	0.87	1.15	0.88	.864	1.75	1.83	-.237**
T4 anger	1.81	0.83	1.15	0.94	.824	1.73	1.84	-.091*
T5 worry	1.90	1.00	1.06	0.64	.960	1.82	1.93	.159**
T5 fear	1.81	0.94	0.99	0.23	.962	1.71	1.86	.151**
T5 boredom	1.77	0.85	1.21	1.16	.833	1.69	1.80	-.247**
T5 anger	1.77	0.84	1.26	1.59	.834	1.67	1.80	-.118**
BIS	16.49	4.48	0.12	-0.57	.770	15.44	16.79**	-.153**
BAS	16.39	3.43	-0.20	-0.19	.720	16.94**	16.23	-.149**
Fight	13.99	3.72	0.25	-0.20	.776	14.72**	13.78	-.075**
Flight	13.56	2.98	-0.26	-0.28	.586	12.49	13.86**	-.122**
Freeze	10.20	3.47	0.48	-0.34	.771	8.71	10.63**	-.066*
Protection	4.22	0.97	-1.21	.859	.713	4.02	4.30**	.049
Media	1.88	0.33	[c]		.692	χ^2 (1) = .11, p > .05		.068[e]
Daily routine	1.86	0.34			.628	χ^2 (1) = .01, p > .05		.150**
Hobby	1.96	0.19			.658	χ^2 (1) = .95, p > .05		-.124**
Study/work from home	1.80	0.40			.824	χ^2 (1) = 4.51, p < .05[d]		-.065

M – mean; SD – standard deviation; Sk – skewness; Ku – kurtosis; R – reliability, represented with Cronbach α for RST dimensions, and with Interclass correlation `coefficient for all other measures; a - t-tests were used to compare gender differences; b - Pearson correlation coefficient; c – skewness and kurtosis values were omitted for binary variables; d - χ2 was used to calculate the association between gender and categorical variable. Females were more frequent in active study/work from home. e – point-biserial coefficient of correlation was computed between age and binary variables; f – single-item measure, measured only in one point, reliability could not be computed. - p < .05; ** - p < .01. ICCworry = .951. ICCfear = .927. ICCboredom = 927. ICCanger = .892.

Appendix C.

Bonferroni Post-hoc tests for five measurement points

Question	Measurement point		Mean Difference	Standard error	p value	95% Confidence	
						Lower CI	Upper CI
Worry	T1	T2	.240*	0.03	0.000	0.16	0.33
		T3	.474*	0.03	0.000	0.38	0.57
		T4	.627*	0.04	0.000	0.52	0.73
		T5	.803*	0.04	0.000	0.69	0.92
	T2	T3	.234*	0.03	0.000	0.15	0.32
		T4	.387*	0.03	0.000	0.30	0.48
		T5	.563*	0.04	0.000	0.46	0.67
	T3	T4	.153*	0.02	0.000	0.09	0.21
		T5	.329*	0.03	0.000	0.25	0.41
	T4	T5	.176*	0.02	0.000	0.12	0.23
Fear	T1	T2	0.04	0.03	1.000	-0.04	0.12
		T3	.131*	0.03	0.000	0.05	0.22
		T4	.246*	0.03	0.000	0.15	0.34
		T5	.379*	0.04	0.000	0.28	0.48
	T2	T3	.092*	0.03	0.005	0.02	0.17
		T4	.207*	0.03	0.000	0.12	0.29
		T5	.340*	0.03	0.000	0.25	0.43
	T3	T4	.115*	0.02	0.000	0.06	0.17
		T5	.248*	0.03	0.000	0.18	0.32
	T4	T5	.133*	0.02	0.000	0.08	0.19
Boredom	T1	T2	.108*	0.03	0.004	0.02	0.19
		T3	.179*	0.04	0.000	0.08	0.28
		T4	.208*	0.04	0.000	0.10	0.31

		T5	.243*	0.04	0.000	0.14	0.34
	T2	T3	0.07	0.03	0.104	-0.01	0.15
		T4	.101*	0.03	0.023	0.01	0.19
		T5	.135*	0.03	0.001	0.04	0.23
	T3	T4	0.03	0.02	1.000	-0.04	0.10
		T5	0.06	0.03	0.253	-0.02	0.14
	T4	T5	0.03	0.03	1.000	-0.04	0.11
Anger	T1	T2	0.01	0.03	1.000	-0.09	0.11
		T3	0.03	0.04	1.000	-0.07	0.14
		T4	0.09	0.04	0.101	-0.01	0.19
		T5	.116*	0.04	0.035	0.00	0.23
	T2	T3	0.03	0.03	1.000	-0.06	0.12
		T4	0.08	0.03	0.104	-0.01	0.18
		T5	.109*	0.04	0.042	0.00	0.22
	T3	T4	0.06	0.03	0.380	-0.02	0.14
		T5	0.08	0.03	0.136	-0.01	0.18
	T4	T5	0.02	0.03	1.000	-0.06	0.11

Appendix D.

Correlation between emotional responses, RSQ dimensions and behaviors related to pandemic

	1	2	3	4	5	6	7	8	9	10	11	12	13	14
Worry (1)														
Fear (2)	.678**													
Boredom (3)	.155**	.083**												
Anger (4)	.315**	.251**	.428**											
BIS (5)	.146**	.177**	.141**	.187**										
BAS (6)	-.099**	-.153**	.033	-.016	-.342**									
Fight (7)	.069*	.036	.118**	.132**	-.093**	.274**								
Flight (8)	.126**	.133**	.101**	.085**	.449**	-.217**	-.117**							
Freeze (9)	.085**	.130**	.117**	.174**	.604**	-.254**	-.228**	.459**						
Protection (10)	.178**	.188**	-.107*	-.029	.042	-.057	-.020	.062	.000					
Media (11)	.251**	.238**	.028	.060	.107**	-.054	.026	.096*	.080*	.088				
Daily routine (12)	-.025	.011	-.201**	-.111**	-.096*	.048	-.013	-.048	-.014	.024	.063			
Hobby (13)	-.116**	-.074	-.023	-.069	-.047	.063	.042	.005	-.061	-.076	.026	.120**		
Study/work from home (14)	-.028	-.009	-.117**	-.027	-.011	.046	-.035	.019	.017	.110*	.013	.215**	.049	

*p < .05
**p < .01
